# Endoscopic Treatment of a Bile Duct Stone Containing a Surgical Staple

**DOI:** 10.1155/1990/53506

**Published:** 1990

**Authors:** J. A. Janson, P. B. Cotton

**Affiliations:** 1 Department of Medicine Division of Gastroenterology Duke University Medical Center Durham N.C. USA; 2 Associate in Medicine Box 3913 Duke University Medical Center Durham N.C. USA

## Abstract

We report a case of a pigmented gallstone which formed around a surgical staple in the bile duct. The stone was removed and retrieved endoscopically. A brief review of bile duct foreign bodies and gallstones is presented.


*HPB Surgery*, 1990, Vol. 3, pp. 67–71
Reprints available directly from the publisher
Photocopying permitted by license only

© 1990 Harwood Academic Publishers GmbH
Printed in the United Kingdom

### CASE REPORT

### ENDOSCOPIC TREATMENT OF A BILE DUCT STONE CONTAINING A SURGICAL STAPLE

J.A. JANSON* and P.B. COTTON
*Department of Medicine, Division of Gastroenterology, Duke University Medical Center, Durham, N.C. USA*

*(Received 18 March 1990)*

We report a case of a pigmented gallstone which formed around a surgical staple in the bile duct. The stone was removed and retrieved endoscopically. A brief review of bile duct foreign bodies and gallstones is presented.

KEY WORDS: Choledocholithiasis, foreign bodies, endoscopic therapy

### CASE REPORT

A 48 year old female with a history of biliary colic underwent uneventful cholecystectomy in 1979 for gallstones. She was well until November 1988 when she presented with several days of nausea, vomiting and abdominal pain. A HIDA Scan indicated complete obstruction of the common bile duct (CBD). Liver function tests (LFT's) were consistent with obstruction and the amylase was significantly elevated. The symptoms subsided spontaneously with conservative management and repeat HIDA scan was normal. The presumptive diagnosis was biliary pancreatitis with spontaneous passage of a stone.

One year later she presented with nausea, excruciating abdominal pain, fever and mild jaundice. Again, HIDA scan showed complete obstruction of the CBD, LFT's were obstructive but the amylase was normal. She had a leukocytosis and blood cultures grew sensitive coliform bacteria. She was treated with ampicillin with a good response. Three days later she was referred to Duke University Medical Center for further evaluation and treatment.

On admission she appeared well with mild biochemical cholestasis. Endoscopy revealed a normal stomach, pylorus, duodenum and ampulla. On fluoroscopy surgical staples were seen in the biliary area. Cannulation of the papilla and cholangiography revealed a dilated bile duct (14mm) with a 14mm filling defect containing a surgical clip (Figure 1). A standard sphincterotomy was performed

*Correspondence to: Dr. J.A. Janson, Associate in Medicine, Box 3913, Duke University Medical Center, Durham, N.C. USA

and the stone was grasped with a retrieval basket (Figure 2). The surgical staple moved with the stone, and upon extraction was seen embedded in it. (Figure 3).

Stone analysis revealed calcium bilirubinate (66%) as the major component with cholesterol (30%), mixed bile pigments (3%) and calcium salts of fatty acids (1%) consistent with a primary pigment stone.

The patient did well and was discharged. At four weeks follow up the patient had no complaints.

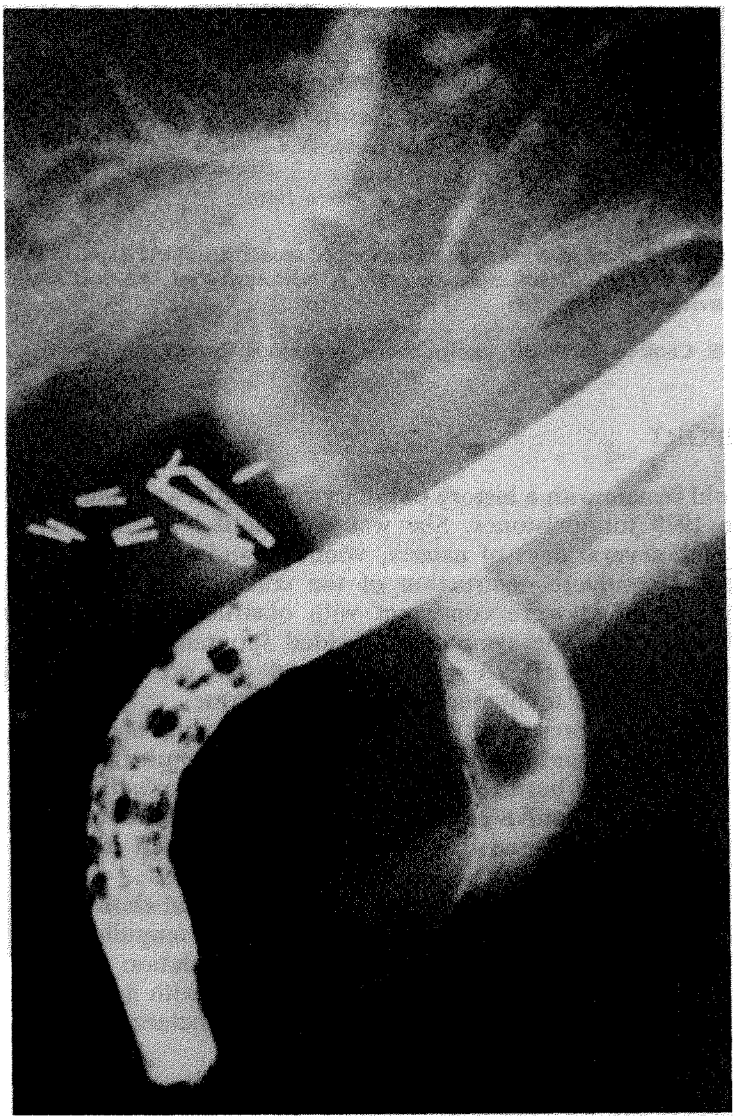

**Figure 1** Cholangiogram showing filling defect and surgical clip in the common bile duct.

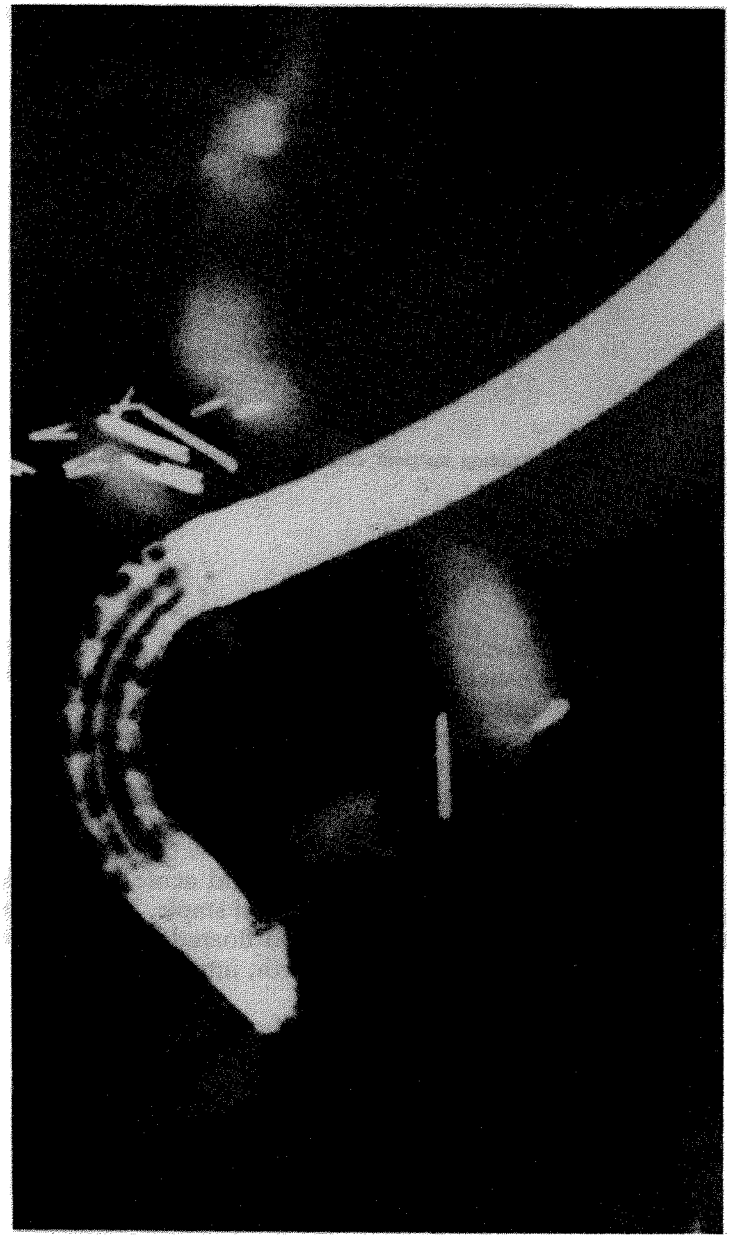

**Figure 2** Cholangiogram showing a retrieval basket grasping the stone/staple.

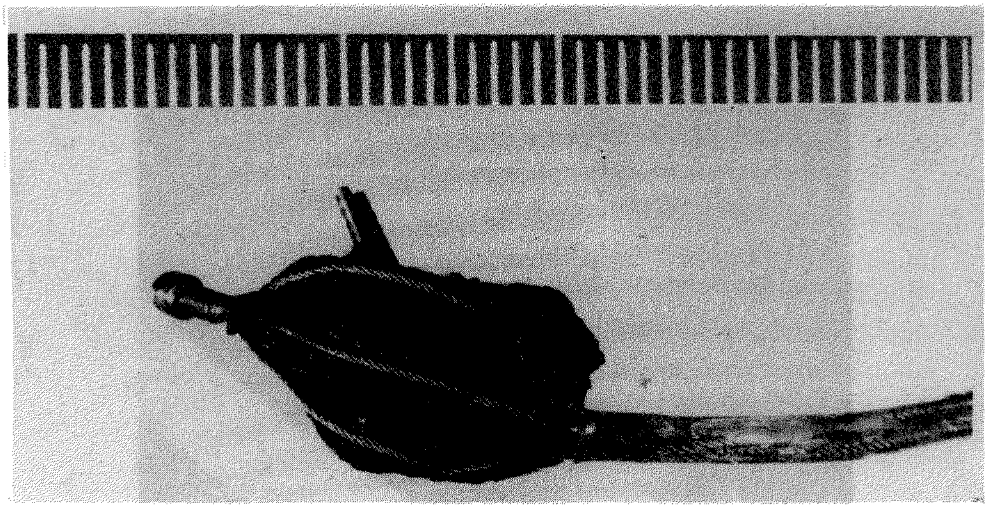

**Figure 3** Gallstone with the protruding surgical clip as removed in the basket. Each hash mark represents 1mm.

### DISCUSSION

Choledocholithiasis after cholecystectomy is a well known phenomenon. Primary pigment stone formation is usually noted years after surgery and is associated with bile stasis and bacterial action of bilirubin forming a glycocalyx for further deposition of stone substrates[1]. Several different foreign bodies have been implicated as providing a nidus for stone formation in the bile duct. Non-absorbable sutures are most commonly seen, but shrapnel[2], buckshot[2], wire fragments[3], prosthetic tubes[4], vegetable residue[4,5], fishbones[6], and surgical clips[7,8,9,10], have also been reported.

Our patient had a history of both pancreatitis and acute cholangitis due to a common duct calculus that formed around a surgical staple. We speculate that the staple eroded through the cystic duct stump and floated into the common duct, providing a nidus for nucleation and stone growth; impaction of the stone caused the episodes of pancreatitis and cholangitis.

Endoscopic sphincterotomy and extraction of calculi is a well accepted, non-surgical alternative treatment for choledocholithiasis [11,12,13]. Foreign body extraction has also been reported[4]. However, this is only the second report of endoscopic extraction of a staple-stone combination[8].

### *References*

1. Stewart, L., Smith, A.L., Pellegrini, C.A., Motson, R.W. and Way, L.W. (1987) Pigment gallstones form as a composite of bacterial microcolonies and pigment solids. *Ann. Surg.* **206**, 242–250.
2. Sanowski, R.A. and Arbaje-Ramirez, G. (1979) Acute cholecystitis secondary to unusual gallstones. *Am. J. Gastroenterol* **71**, 193--195

3. Morse, L.J. and Millin, J. (1971) Gallstone formation secondary to a foreign body. *NEJM* **284**, 590–591
4. Bedogni, G. *et al.* (1986) Foreign bodies of the biliary tract, endoscopic management. *Dig. Dis. Sci.* **31**, 1100–1104
5. Conroy, B. and Metcalf, M.J. (1982), Choledocholithiasis associated with plant material in the common bile duct. *J. R. Coll. Surg. Edinb.* **27**, 244–5
6. Orda, R., Leviav, A., Ratan, I., Stadler. J. and Wiznitzer, T. (1986) Common bile duct stone caused by a foreign body. *J.Clin. Gastroenterol* **8**, 466–8
7. Walker, W.E., Avant, G.R. and Reynolds. V.H. (1979) Cholangitis with a silver lining. *Arch.Surg.* **114**, 214–215.
8. Brutran, F.M., Kampschroer, B.H. and Parker, H.W. (1982) Vessel clip as a nidus for formation of common bile duct stone. *Gastrointest. Endosc.* **28**, 222–223
9. Margolis, J.L. (1986) Recurrent choledocholithiasis due to hemostatic clip. *Arch. Surg.* **121**, 1213
10. Davis, M., Hart, B. and Kleinman, R. (1988) Obstructive jaundice from open vessel clip. *Gastrointest Radiol.* **13**, 259–260
11. Allen, B., Shapiro, H. Way, L.W. (1981) Management of recurrent and residual common duct stones. *Am. J. Surg.* **42**, 41
12. Cotton, P.B. (1984) Endoscopic Management of bile duct stones (Apples and Oranges) *Gut* **25**, 587–597
13. Classen, M. (1987) Endoscopic papillotomy. In Sivak, M.J, Jr., Gastroenterologic Endoscopy, Philadelphia, W.B. Saunders, p631–651

*(Accepted by John L. Cameron March 28 1990)*

